# Genome wide DNA differential methylation regions in colorectal cancer patients in relation to blood related family members, obese and non-obese controls – a preliminary report

**DOI:** 10.18632/oncotarget.25374

**Published:** 2018-05-22

**Authors:** S. Pamela K. Shiao, Haiyan Xiao, Lixin Dong, Xiaoling Wang, Kebin Liu, Jinxiong She, Huidong Shi

**Affiliations:** ^1^ College of Nursing, Augusta University, Augusta, GA, USA; ^2^ Medical College of Georgia, Augusta University, Augusta, GA, USA; ^3^ Center for Biotechnology and Genomic Medicine, Augusta, GA, USA; ^4^ Georgia Prevention Institute, Augusta, GA, USA; ^5^ Georgia Cancer Center, Augusta, GA, USA

**Keywords:** Genome wide methylation, DNA methylation regions, CRC, blood biomarkers

## Abstract

Despite evidences linking methylation changes in the cancer tissues, little is known about the methylation modification in the peripheral blood. With the current study, we identified differential methylation regions (DMRs) across human genome by collecting the blood samples of colorectal cancer (CRC) patients compared to that of their blood-related family who shared genetic inheritance and environmental influences, and unrelated obese and non-obese controls by accessing publicly available Gene Expression Omnibus data. We performed genome-wide analyses using the reduced representation bisulfite sequencing (RRBS) method covering about 25% of CpGs for whole human genome of the four groups (n = 5 each). In comparison to the non-obese controls, we observed significant DMRs in CRC for genes involved in tumorigenesis including *MLH3, MSH2*, *MSH6, SEPT9, GNAS*; and glucose transporter genes associated with obesity and diabetes including *SLC2A1/GLUT1,* and *SLC2A3/GLUT3* that were reported on methylation being modified in cancer tissues. In addition, we observed significant DMRs in CRC for genes involved in the methylation pathways including *PEMT*, *ALDH1L1*, and *DNMT3A*. CRC and family members shared significant DMRs for genes of tumorigenesis including *MSH2*, *SEPT9, GNAS, SLC2A1/GLUT1 and SLC2A3/GLUT3*); and *CAMK1, GLUT1/SLC2A1* and *GLUT3/SLC2A3* genes involved in glucose and insulin metabolism that played vital role in development of obesity and diabetes. Our study provided evidences that these differentially methylated genes in the blood could potentially serve as candidate biomarkers for CRC diagnostic and may provide further understanding on CRC progression. Further studies are warranted to validate these methylation changes for diagnostic and prevention of CRC.

## INTRODUCTION

Colorectal cancer (CRC) is the third most common cancer among both men and women, and the third most common cause of cancer-related deaths [1], for past 4 decades in the United States. For 2018, about 97,220 newly diagnosed CRC cases and 50,260 CRC-related deaths were projected [2]. Genetic mutations and epigenetic modifications in oncogenes and/or tumor suppressor genes cause the development of cancer [3]. Given that DNA methylation is one of the most important epigenetic events, the identification of CRC-specific methylation markers may provide new insights for better understanding of CRC progression and early cancer detection.

Methylation is a reversible attachment of a methyl group to a CpG dinucleotide, rendering it not being able to be transcribed, affected by both genetic and environmental factors [4–6]. In the past decade, it has become clear that cancer cells have aberrant patterns of DNA methylation at the individual CpG sites or a group of CpG sites in close proximity, which are denoted as differentially methylated cytosines (DMCs) and differentially methylated regions (DMRs), respectively [7–9]. For example, it has been reported that some genes are hyper-methylated at the promoter region in CRC including *APC*, *MGMT* and hMLH1 (see Supplementary Table 1 for the list of genes, full names, and gene functions presented in this paper). Additionally, significant associations were documented between DNA methylation and cancer progression, such as changes of DMR at the promoter region of *RASSF1A* gene in association with tumor stages [8]. Additionally, hyper-methylation at the promoter regions of *SYNE1* and *FOXE1* genes was presented in colitis-associated colorectal neoplasia [9]. Most of those studies were focused on tumor tissues to explore the associations between DNA methylation status and CRC as potential biomarkers. Tumor tissue collection would involve invasive approach which is not as readily accessible compared to other less invasive methods such as blood-based detection of CRC. Studies using blood-based methods, however, have relied on testing a limited number of pre-selected genes and on the use of non-quantitative detection methods, such as gel-based methylation-specific polymerase chain reaction (PCR). In the current study we identified different methylation patterns across the whole human genome covering about 25% of CpGs for whole human genome (see Method section), using the blood samples of CRC patients compared to other controls, with the reduced representation bisulfite sequencing (RRBS) method.

Dietary habits, life-styles and environmental agents contribute to epigenetic changes [10]. For example, a diet rich in polyunsaturated fatty acids could generate mutagenic free radicals and oxidative stress to cause epigenetic alterations [11]. Folate metabolism which also involves Vitamin B12 (both important factors in the one-carbon metabolism pathway), provides the one-carbon units required for intracellular processes, including the synthesis of S—adenosylmethionine (SAM) which is required for DNA methylation and synthesis [12]. Besides genetic inheritance, family members tend to share similar dietary habits, life-styles, and the exposure to the environmental agents. These could result in common epigenetic alterations among the family members and within the family units. However, those probable common signatures among CRC and family members were under studied. In this study, in addition to the CRC cases, we included their blood-related family members living in the same household with the cases who shared not only genetic inheritance but also environmental influences, to examine genome wide methylation profiles of their blood.

Further studies have revealed a risk association for obesity and CRC [13]. Moreover, many studies presented the effects of adiposity on individual genes or methylation processes [14–17]. Adiposity interfered with age-induced epigenetic changes in methylation studies [15] and affected regulatory processes in epigenetic pathways [16]. Obese males are known to have an increased risk of developing CRC compared to non-obese subjects [17]. However, how the methylation processes affect gene ontology for metabolic pathways of CRC is not well documented. Therefore, we included the RRBS based methylation data of obese and non-obese people compared with the data of CRC cases and their blood-related family members.

In summary, with the current study, we analyzed genome wide methylation profiles using blood samples of CRC cases, their blood-related family members, also unrelated obese and non-obese controls. The findings from this study could advance our understanding of how shared genetic inheritance and life experiences within the blood-related family units and adiposity as environmental influences affecting the metabolic processes and epigenetic mechanisms of CRC, which might provide a better insight to the diagnosis, treatment, and prevention of CRC.

## RESULTS

### Participants

The demographic characteristics and CpG methylation data of the four groups are presented in Table 1. The subjects included 5 CRC patients and their blood-related family members who shared genetic heritage and same household without cancer from a family-based study. Data included RRBS data for these 5 CRC cases and 5 family members, and RRBS data of 5 obese subjects and 5 matched non-obese controls, based on age and gender with the available data, downloaded from the Gene Expression Omnibus (GEO) data that is available to the public (https://www.ncbi.nlm.nih.gov/geo/query/acc.cgi?acc=GSE85928, see Method section for additional details).

**Table 1:** Sample characteristicsand CpG sequencing data of the four groups

n mean ± SD	Control n=5	Obesity n=5	Family n=5	Cancer n=5
Gender				
Female	3	2	2	5
Male	2	3	3	0
BMI				
≤ 30	5	0	4	3
>30	0	5	1	2
Age	35 ± 7	45 ± 5	31 ± 13	58 ± 8
Raw reads	34,560,581 ± 17,900,576	28,087,864 ± 3,628,196	46,728,944 ± 5,939,651	34,857,916 ± 5,649,474
QC-passed reads	24,300,490 ± 12,874,723	19,538,524 ± 2,733,127	16,917,641 ± 1,803,606	12,528,537 ± 1,986,199
Mapping Efficiency	69.9 ± 1.44	69.52 ± 2.31	72.56 ± 1.95	71.93 ± 0.88
# of CpGs *> 0 read*	6,328,477 ± 811,790	6,436,231 ± 697,978	7,458,949 ± 494,165	6,632,163 ± 726,277
# of CpGs *> 5 read*	4,184,837 ± 479,512	4,291,259 ± 242,916	4,037,734 ± 415,253	3,669,350 ± 342,216
CpG Methylation *%*	41.7 ± 0.63	41.51 ± 1.00	45.69 ± 2.07	42.6 ± 2.87
CpG Coverage	19.60 ± 10.06	15.27 ± 2.84	9.53 ± 1.14	9.60 ± 2.36
# of CGI	24,268 ± 323	24,294 ± 348	24,483 ± 143	24,186 ± 236
# of CpGs on CGI	2,186.183 ± 281,350	2,195,293 ± 254,964	2,324,162 ± 44,814	2,266,921 ±77,206

Note. BMI: body mass index; QC: quality control; CGI: CpG Island.

### Genome-wide bisulfite sequencing

To perform a genome-wide analysis of DNA methylation in CRC and family control, in comparison to the obese and non-obese controls, we generated 20-50 million Illumina sequencing reads for each sample yielding an average of 6.6 to 7.5 million CpGs which covered 23.2 – 26.5% of CpGs for whole human genome (28.3 million CpGs total) [18, 19] (Table 1). Of these, 62% to 73% were successfully mapped to either strand of the human genome (hg19). The average sequencing depth per CpG was between 7x and 35x. We were able to determine the methylation status of approximately 1.7–2.3 million CpGs on CpG Islands (CGI) (Table 1).

### Differentially methylated region (DMR) analysis in 6 regions

**Figure 1:**
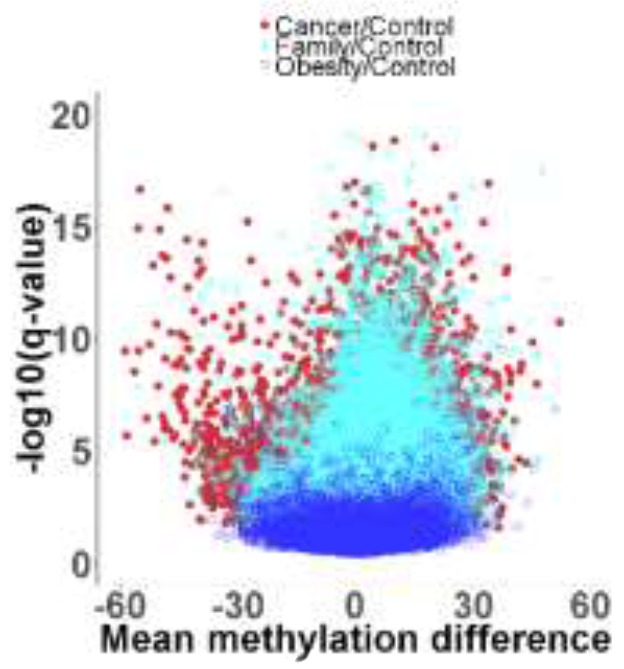
Volcano plot showing adjusted q values versus mean methylation difference between groups of Cancer (red color), Family (teal color), and Obese (blue color) with non-obese Control

To identify the DMRs between one of the three groups [CRC (Cancer), blood-related family members of CRC patients (Family), and obese] with non-obese control, we then performed a genome-wide unbiased DMR detection using a complete tiling of the human genome in 200 bp windows. Figure 1 is a volcano plot showing the adjusted q-values for all DMRs versus mean methylation difference between each of the three groups and the non-obese control group. Using the criteria requiring an adjusted q value < 0.05 and difference of average methylation level > 5%, 10% or 15%, we identified different % DMRs in each of the three groups when compared with non-obese controls (Table 2 and Supplementary Figure 1). Using a 5% differential methylation level criterion, we identified a total of 11,866 hyper-methylated DMRs for the Cancer/control pair, 12,700 hyper-methylated DMRs for the Family/control pair, and 2404 hyper-methylated DMRs for the obese/control pair. Whereas, when a 10% differential level was used, 4876, 5085, and 1291 hyper-methylated DMRs per pair were identified; and, with a 15% differential level, 1865, 1784, and 592 hyper-methylated DMRs were noted for the three pairs (Table 2). There were decreased numbers of DMRs as the % differential levels increased. Additionally, there were 4591 hypo-methylated DMRs for the Cancer/control pair, 2235 hypo-methylated DMRs for the Family/control pair, and 1961 hypo-methylated DMRs for the obese/control pair, when 5% differential was used as the criterion. The numbers were 2451, 987, and 912 respectively using a 10% differential criterion; and 1308, 449, and 376 of hypo-methylated DMRs respectively for a 15% criterion.

**Table 2:** Number of hyper- and hypo- differential methylated regions (DMRs) per grouping comparisons at 5%, 10%, and 15% differences

Groups	Cancer/Control		Family/Control		Obese/Control	
Differential Methylation	Hyper-	Hypo-	Hyper-	Hypo-	Hyper-	Hypo-
5%	11,866	4591	12,700	2235	2404	1961
10%	4876	2451	5085	987	1291	912
15%	1865	1308	1784	449	592	376

Overall, there is a trend that more DMRs were presented for the Cancer/control compared to the Family/control, and least DMRs for the obese/control pair, indicating that there were more changes on the methylation for Cancer than the Family and obese groups compared to the non-obese controls. Those results also suggested that genetic and life style that were shared by the blood-related family members might be involved in methylation modifications as more DMRs were found for the Family/control pair compared to the obese/control pair (Table 2 and Supplementary Figure 1). By using a 10% differential criterion as the methylation difference, we identified the distribution of DMRs in each of the genome regions. Supplementary Table 2 presents that about 5-7% of the DMRs were presented in the promoter region. 30% of the DMRs in the Intron region, and 40% of the DMRs in the intergenic or intragenic regions. The results on these percent distributions were comparable for the three paired group comparisons.

### Top 10 methylated DMRs-genes: Cancer versus control pair

Table 3 lists the top 10 most significant hyper-methylated DMRs (distance to transcription start site [TSS] were from -1,000 to +1,000 base-pair [bp] DNA) for Cancer/control pair, and the annotated genes are *PCNXL3, MIR4285, NLGN2, MIR3648, HOXA4, CLDN23, TONSL, GNAS, TUBB8* and *MIR1247*. Table 4 lists the top 10 most significant hypo-methylated DMRs for Cancer/control pair (Distance to TSS are from -1,000 to +1,000 bp). The annotated genes are *SLC2A3 (GLUT3), LOC338817, MLH3, LRRC27, FANCG, RPSA, SLC2A1 (GLUT1), ZFP36, HMHA1*, and *ARID5B*. Many of those genes had been shown to be differently expressed in CRC tumor tissues, that were also presented in our study using the blood samples. Thus, those methylation changes could be potential biomarkers in the blood for CRC diagnostic or provide further understanding on CRC development. It is worthy to notice that half of the hyper-methylated DMRs were in the DNA coding sequence (CDS) regions as compared to the other half in other combination of promoter, five prime untranslated (Utr5) and intron regions; whereas, half of the hypo-methylated DMRs were in the promoter regions as compared to the other half being the combination of intron, CDS, and Utr5 regions.

**Table 3:** Top 10 hyper-methylated DNA methylated regions based on 10% difference between Cancer and Control (-1000 ≤ distance to transcription start site [TSS] ≤ +1000 base-pair DNA)

Gene	Regions	DMR location		Distance To TSS	Methylation Difference			Gene Name or Role
		Start	End		%	*p*	*q*	
*PCNXL3*	CDS	65,402,926	65,403,007	-774	39.49	**3.20E-13**	1.63E-11	Homeostasis/metabolism phenotype
*MIR4285*	Utr5	101,936,380	101,936,461	**13**	38.53	4.00E-07	2.85E-06	MicroRNA 4285
*NLGN2*	CDS	7,311,698	7,311,857	198	34.28	9.29E-05	2.72E-04	Mediates cell-cell interactions and modulates insulin secretion
*MIR3648*	Utr5	9,825,819	9,826,028	0	33.93	5.21E-21	3.53E-18	MicroRNA 3648
*HOXA4*	CDS	27,169,637	27,170,022	378	31.68	2.04E-03	3.40E-03	Sequence-specific DNA binding transcription factor activity
*CLDN23*	CDS	8,560,299	8,560,499	635	31.19	1.56E-04	4.17E-04	Structural molecule activityidentical protein binding
*TONSL*	CDS	145,661,276	145,661,402	-275	31.03	3.44E-10	7.12E-09	Transcription corepressor activity
*GNAS*	Promoter	57,464,802	57,465,121	624	28.79	3.48E-04	8.05E-04	Insulin-like growth factor receptor binding
*TUBB8*	Intron	95,010	95,073	106	27.79	4.90E-04	1.07E-03	Structural constituent of cytoskeleton
*MIR1247*	Promoter	102,028,543	102,0l28,738	857	27.63	5.44E-05	1.75E-04	MicroRNA 1247

Note: CDS: coding DNA sequence; Utr5: five prime untranslated.

**Table 4:** Top 10 hypo-methylated DNA methylated regions based on 10% difference between Cancer and Control (-1000 ≤ distance to transcription start site [TSS] ≤ +1000 base-pair DNA)

Gene	Regions	DMR location		Distance To TSS	Methylation Difference			Gene Name or role
		Start	End		%	*p*	*q*	
*SLC2A3*	Intron	8,087,820	8,087,905	988	-61.18	1.06E-13	6.23E-12	Glucose transmembrane transporter activity
*LOC338817*	Promoter	11,700,194	11,700,609	-355	-55.46	1.36E-18	3.92E-16	N/A
*MLH3*	Promoter	75,518,893	75,518,943	-659	-51.32	2.22E-07	1.73E-06	DNA Mismatch Repair (MMR)
*LRRC27*	Promoter	134,144,308	134,144,429	951	-49.30	1.06E-08	1.32E-07	Leucine Rich Repeat Containing 27
*FANCG*	Promoter	35,080,807	35,080,913	-795	-48.79	2.24E-08	2.51E-07	DNA Double-Strand Break Repair
*RPSA*	Intron	39,450,361	39,450,501	481	-46.51	1.53E-07	1.27E-06	RNA binding, structural constituent of ribosome
*SLC2A1*	Promoter	43,425,368	43,425,715	-522	-44.71	8.39E-10	1.51E-08	Glucose transmembrane transporter activity
*ZFP36*	CDS	39,899,297	39,899,354	965	-43.30	2.97E-06	1.56E-05	DNA binding, RNA binding, Protein binding
*HMHA1*	Intron	1,077,542	1,077,680	911	-42.82	3.24E-12	1.23E-10	Minor histocompatibility protein HA-1
*ARID5B*	Utr5	63,808,928	63,809,171	0	-42.54	6.81E-07	4.50E-06	FTO Obesity Variant Mechanism

Note: CDS: coding DNA sequence.

### Top 10 methylated DMRs-genes: Family versus control pair

Top 10 hyper-methylated DMRs for Family/control pair are listed in Supplementary Table 3, the annotated genes are ***PCNXL3***, *RFPL2, LOC729176,* ***TONSL***, ***NLGN2***, ***GNAS****, TPRX1, EGFLAM, PRKAR1B*, and ***MIR3648*** (genes underlined are overlapping with genes found for the Cancer/control pair in Table 3). Supplementary Table 4 lists top 10 hypo-methylated DMRs for Family/control, and the related genes are *SLC2A3, LOC338817,* ***SLC2A1 (GLUT1)****, METTL16, SEPT9, MEG3, HMHA1, HOXB6, CPOX*, and ***SLC2A3 (GLUT3)*** (genes underlined are overlapping with genes found for the Cancer/control pair in Table 4). Interestingly, among those top 10 hyper- and hypo- methylated genes, several of them are overlapping with those in Cancer/control pair, including *PCNXL3, TONSL, NLGN2, GNAS, MIR3648, SLC2A3 (GLUT3) and SLC2A1 (GLUT1)*. This finding supports the hypothesis that blood-related family members shared similar epigenetic modifications with CRC patients, as they shared genetic heritage and might have also shared similar dietary habits, life style, and environmental agents in the same household in addition to their genetic makeup. It is worthy to notice that hyper-methylated DMRs were about evenly distributed in the CDS, promoter, intron and Utr5 regions; whereas, half of the hypo-methylated DMRs were in the promoter, and other half in intron regions (40%), and finally in Utr5 regions.

### Top 10 methylated DMRs-genes: Obesity versus control pair

Supplementary Table 5 presents top 10 hyper-methylated DMRs for obese/control pair, and the genes are *RGPD5/RGPD8, CTDSPL2, GCNT1, LMO2, PGPEP1L, LDHA, CYB5R2, SPACA1, PARVG*, and *MSH6*. Top 10 hypo-methylated DMRs for obese/control are listed in Supplementary Table 6. The annotated genes are *LHX6, INPP5F, HIGD1A,* ***SLC2A3 (GLUT3)****, BLCAP, NNAT, MATR3, SNHG4, CCDC144B*, and *DTX1* (gene underlined is also found for the Cancer versus control pair in Table 4). And, *SLC2A3* was also on the top 10 hypo-methylated gene list in Cancer/control pair comparison, which suggested some association of obesity and CRC. It is worthy to notice that hyper-methylated DMRs are about evenly distributed in the CDS, promoter, intron and Utr5 regions; whereas, more regions of the hypo-methylated DMRs are in the promoter (40%), than intron (30%) and other three regions of CDS, Utr5 and three prime untranslated (Utr3) regions (10% each).

### Targeted genes

**Figure 2:**
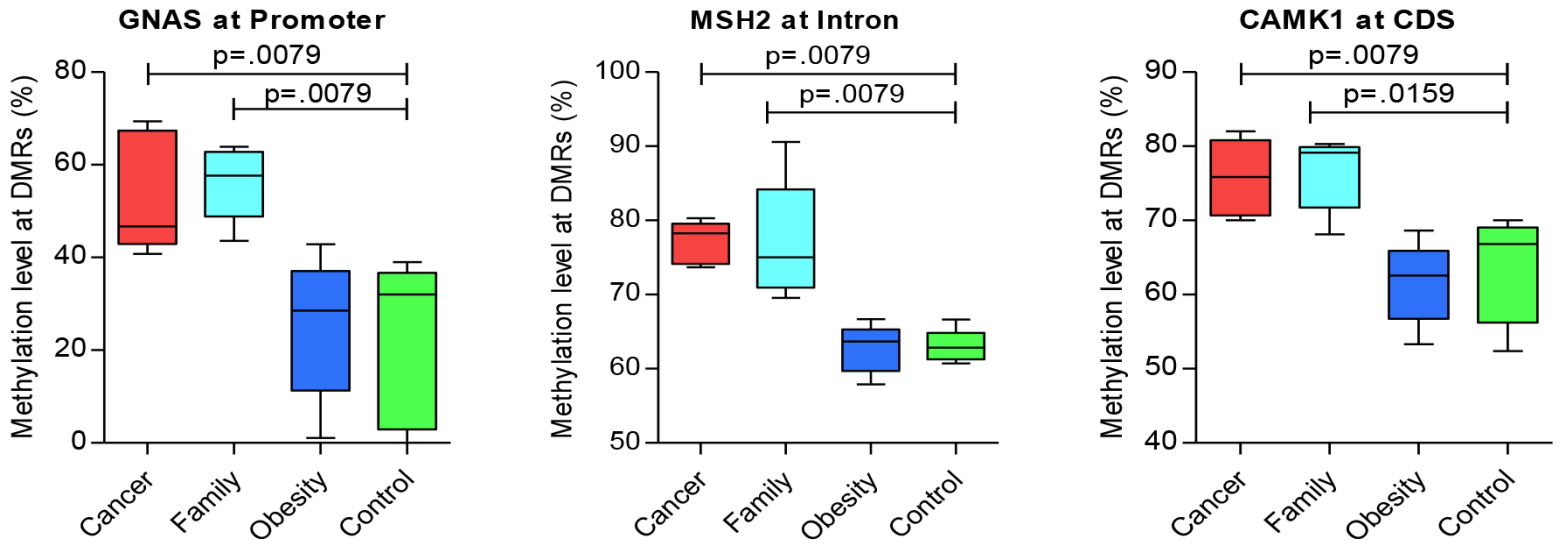
Significant genes of hyper-methylation compared between groups (CDS: coding DNA sequence region)

**Figure 3:**
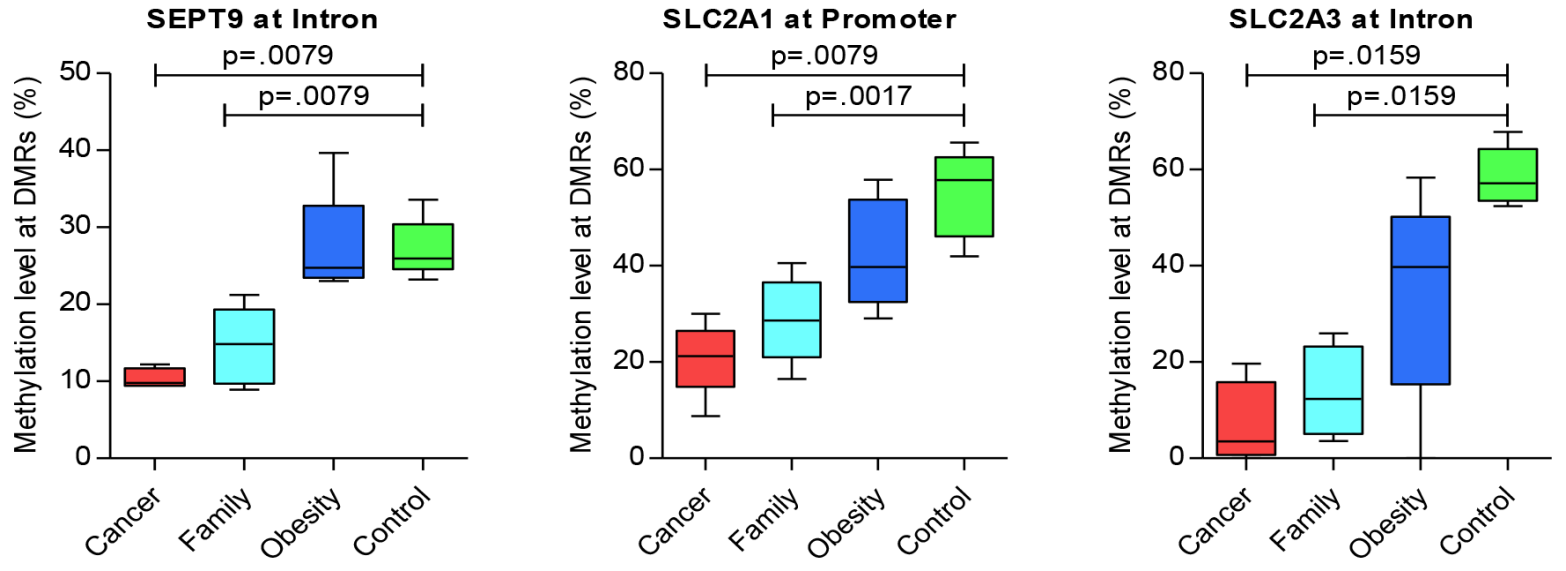
Significant genes of hypo-methylation compared between groups

For additional genes of significance between Cancer versus control groups, Table 5 presents hyper-methylated DMRs, with Figure 2 presenting the three most representative genes, ***GNAS, MSH2***, and ***CAMK*** that were significantly different for Case/control and Family/control pairs, and Supplementary Figure 2 presenting additional genes of significance for Cancer/control pair. And, Table 6 presents significant hypo-methylated DMRs and associated genes that showed difference between Cancer versus control groups, with Figure 3 presenting the three most representative genes, ***SEPT9***, ***SLC2A1/GLUT1*** *and* ***SLC2A3/GLUT3***, that were significantly different for both Cancer/control and Family/control pairs, and Supplementary Figure 3 presenting additional genes of significance for Cancer/control pair.

**Table 5:** Significant hyper-methylated genes based on 10% methylation difference between Cancer and Control (-1000 ≤ distance to transcription start site [TSS] ≤ +1000 base-pair DNA)

Gene	Regions	DMR location		Distance To TSS	Methylation Difference			Gene Role
		Start	End		%	*p*	*q*	
*GNAS*	Promoter	57,464,802	57,465,121	624	28.79	3.48E-04	8.05E-04	GNAS mutation associated with colorectal tumorigenesis [30]
	Promoter	57,425,903	57,426,055	0	26.79	1.59E-06	9.22E-06	
*DNMT3A*	Intron	25,551,093	25,551,226	365	23.78	8.39E-07	5.36E-06	Methylation pathway
*MSH6*	Intron	48,011,362	48,011,896	270	20.76	2.71E-03	4.28E-03	Lynch Syndrome, MMR
*CAMK1*	CDS	9,799,262	9,799,361	7,636	15.32	1.06E-04	3.03E-04	Calcium/calmodulin-dependent protein kinase type 1
*MSH2*	Intron	47,660,208	47,660,258	30,004	14.75	1.08E-04	3.07E-04	Lynch Syndrome, MMR
*GNAS*	Intron	57,416,524	57,416,680	0	14.50	9.23E-04	1.79E-03	Associated with colorectal tumorigenesis
*HK3*	CDS	176,308,803	176,309,092	17,242	14.45	3.26E-05	1.16E-04	Glucose metabolism pathways
*GNAS*	Promoter	57,426,743	57,427,047	-786	13.72	6.80E-03	9.05E-03	GNAS mutation associated with Cancer
*FCN1*	CDS	137,804,570	137,805,004	4,803	11.72	7.28E-05	2.23E-04	Associated with Diabetes
*HK3*	CDS	176,314,364	176,314,772	11,562	11.32	1.07E-02	1.31E-02	Glucose metabolism pathways
*NOS3*	CDS	150,710,282	150,710,719	10,868	10.69	2.45E-02	2.66E-02	Methylation, oxidative stress

Note: CDS: coding DNA sequence.

**Table 6:** Significant Genes based on 10% differences of methylation level between Cancer and Control (hypo-methylated genes)

Gene	Regions	DMR location		Distance To TSS	Methylation Difference			Gene Role
		Start	End		%	*P*	*q*	
*SLC2A3*	Intron	8,087,820	8,087,905	988	-61.18	1.06E-13	6.23E-12	Glucose transporter 3 (GLUT3)
*MLH3*	Promoter	75,518,893	75,518,943	-659	-51.32	2.22E-07	1.73E-06	DNA mismatch repair genes
*SLC2A1*	Promoter	43,425,368	43,425,715	-522	-44.71	8.39E-10	1.51E-08	Glucose transporter 1 (GLUT1)
*FCN1*	Intron	137,802,212	137,802,301	7,506	-28.36	6.39E-04	1.33E-03	Associated with Diabetes
*SEPT9*	Intron	75,449,408	75,450,396	0	-27.68	2.50E-09	3.82E-08	Tumor suppressor gene
*PEMT*	Intron	17,410,253	17,410,397	-10,545	-23.01	9.47E-05	2.77E-04	Methylation pathway
*ALDH1L1*	Intron	125,832,461	125,832,876	9,980	-11.08	9.75E-04	1.86E-03	Methylation pathway

Figure 2 displays the significant hyper-methylated DMRs with the specific genes. The data reflects an increase in the methylation levels of some DMRs incrementally from control, obese, and Family, to Cancer groups. Pair-wise tests presented significant (*p* < 0.05) hyper-methylated genes between Cancer/control and Family/control pairs, on ***GNAS*** at the promoter region, ***MSH2*** at the Intron region, and ***CAMK2*** at the CDS region (Figure 2). Additional genes with significant trends for group differences for hyper-methylaton included *HK3, FCN1,* and *NOS3* at the CDS region; and, *DNMT3A* and *MSH6* at the intron region (Supplementary Figure 2). Those results suggested common methylation alterations exist in CRC and their family members by sharing genetic heritage, household environment, and epigenetic changes.

Figure 3 presents the significant hypo-methylated DMRs with the specific genes. The data reflects a decrease in the methylation levels of some DMRs from control, obese, and Family to Cancer groups. Pair-wise tests presented significant (*p* < 0.05) hypo-methylated genes between Cancer/control and Family/control pairs on ***SEPT9*** at the intron region, ***SLC2A1/GLUT1*** at the promoter region, and ***SLC2A3/GLUT3*** at the intron region (Figure 3). Additional genes with significance or significant trends for Cancer/control group differences included *FCN1* and *PEMT* at the intron region (Supplementary Figure 3). Those results suggested common methylation alterations exist in CRC and their family members by sharing genetic heritage, household environment, and epigenetic changes. On the other hand, it also suggested that obesity might be associated with CRC as the methylation levels of some of these genes for the obese group are in the mid-range between the CRC and control groups (Figures 2 and 3, Supplementary Figures 2 and Supplementary Figure 3).

Five of these six significant genes shared by Cancer/control and Family/control pairs presented in Figures 2 and 3, in addition to other genes play significant roles in cancer development or tumorigenesis (e.g. ***MLH3, MSH2, MSH6, SEPT9, GNAS, SLC2A1/GLUT1*** *and* ***SLC2A3/GLUT***) [20–22]. *MSH2* and *MSH6* are DNA mis-match repair (MMR) genes for family based hereditary CRC], Lynch Syndrome. Variations in those genes markedly increases the risk of developing Lynch Syndrome [20–22] (also named hereditary nonpolyposis colorectal cancer, HNPCC), which is an inherited disorder that increases the risk of many types of cancer including CRC. *MLH3*is also a MMR, which also plays an important role in HNPCC [23]. The role of **SLC2A1/*GLUT1*** and ***SLC2A3/ GLUT3*** has been widely studied in cancers [24–27]. Researchers summarized that ***GLUT1*** was associated with poor prognosis for disease-free survival in rectal cancer and an indicator of aggressive clinical features in CRC through a meta-analysis [28]. ***SEPT9*** has been reported as being a promising biomarker for early detection and screening of CRC [29, 30], which is also presented in our results using the blood samples. *HK3, FCN1,* ***CAMK1, GLUT1/SLC2A1*** and ***GLUT3/SLC2A3*** are involved in glucose and insulin metabolism that played vital role in development of obesity and diabetes. *FCN1*gene is associated with an earlier onset of type 1 diabetes mellitus in children and adolescents [31]. *PEMT, ALDH1L1, DNMT3A* are critical genes in the methylation pathways. *DNMT3A* is also reported as one of the critical tumor suppressor genes, has crucial biological role in self-renewing cells, enabling their differentiation. Its dysregulation could result in a predisposition to cancer and other pathological consequences [32].

### Gene ontology analysis

For gene ontology analysis, we used 15% differences of average methylation level as the criterion on the DMRs. For Cancer/control pair, from 3173 DMRs (adjusted *q* < 0.05), we identified 1778 known genes. Those genes were uploaded onto the Database for Annotation, Visualization and Integrated Discovery (DAVID) (https://david.ncifcrf.gov), National Institutes of Health [33–35], for Gene Ontology analysis to study whether some common functional trends in *pathways, biological processes, cellular component* and *molecular functions* were associated with those genes. Table 7 lists the Kyoto Encyclopedia of Genes and Genomes (KEGG) PATHWAY and Gene Ontology (GO) categories from DAVID with both raw *p* value and *Benjamini* value to correct for false discovery rate (FDR), of less than 0.05.

**Table 7:** Gene Ontology (GO) analysis based on 15% differences of methylation level between groups (significant according to Benjamin adjusted *p*)

Group/Term	N Genes^*^	*p*, Benjamini
**Cancer/Control**		
Pathways		
hsa04360: Axon Guidance	27	9.59E-06,.00261
hsa05200: Pathways in Cancer	53	3.21E-04,.0429
hsa04015: Rap1 Signaling Pathway	33	4.20E-04,.0375
hsa04510: Focal Adhesion	32	6.40E-04,.0428
hsa04724: Glutamatergic Synapse	21	8.63E-04,.046
Biological Process		
GO:0007156∼Homophilic Cell Adhesion via Plasma Membrane Adhesion Molecules	62	1.21E-27, 4.99E-24
GO:0007165∼Signal Transduction	138	3.71E-07, 7.65E-04
GO:0043547∼Positive Regulation of GTPase Activity	74	1.12E-05, .0153
GO:0050885∼Neuromuscular Process Controlling Balance	14	3.22E-05, .0326
GO:0007155∼Cell Adhesion	61	4.83E-05, .0391
GO:0007399∼Nervous System Development	43	5.17E-05, .035
GO:0016477∼Cell Migration	30	5.97E-05, .0346
Cellular Component		
GO:0005886∼Plasma Membrane	397	4.88E-08, 3.30E-05
Molecular Function		
GO:0005509∼Calcium Ion Binding	119	1.45E-15, 1.76E-12
Family/Control		
Molecular Function		
GO:0005516∼Calmodulin Binding	28	3.88E-06,.00392
Obesity/Control		
Biological Process		
GO:0007156∼Homophilic Cell Adhesion via Plasma Membrane Adhesion Molecules	32	1.04E-17, 2.30E-04
Cellular Component		
GO:0005886∼Plasma Membrane	158	3.61E-06,.0014
Molecular Function		
GO:0005509∼Calcium Ion Binding	51	3.60E-09, 2.29E-06

^*^ Red fonts denote repeated components between the pairs of comparisons.

For the KEGG Pathway analysis, five *pathways* survived FDR for multiple testing corrections, including Axon Guidance (hsa04360), Pathways in Cancer (hsa05200), Rap1 Signaling Pathway (hsa04015), Focal Adhesion (hsa04510), and Glutamatergic Synapse (hsa04724). It has been reported that Axon Guidance gene could turn off tumor suppressor gene in CRC which explains why this pathway survived in Cancer/control pair comparative analysis [36]. Pathways in Cancer, Rap1 Signaling Pathway, and Focal Adhesion were all reported to being involved in cancer development [37–39]. Researchers showed that Rap1 Signaling Pathway played an important role in regulating tumor cell invasion and metastasis [40]. Dysregulated Glutamatergic Signaling Pathways were found as being a player in brain tumor and melanoma [41]. Therefore, all five observed *pathways* are consistent with the cancer status when compared to the healthy non-obese control data.

We also observed 7 *biological processes* that exhibited differences between Cancer and control. Those process includes Hemophilic Cell Adhesion via Plasma Membrane Adhesion Molecules (GO: 0007156), Signal Transduction (GO: 0007165), Positive Regulation of GTPase Activity (GO: 0043547), Neuromuscular Process Controlling Balance (GO: 0050885), Cell Adhesion (GO: 0007155), Nervous System Development (GO: 0007399), and Cell Migration (GO: 0016477). There was one *cellular function* and one *molecular function* survived FDR for multiple testing, named Plasma Membrane (GO: 0005886) and Calcium Ion Binding (GO: 0005509).

For Family/Control pair, from 2233 DMRs (adjusted*q* < 0.05), we identified 1210 known genes. GO analysis was performed using those genes and DAVID database. However, only one *molecular function* showed significant difference between Family/control pair, which is Calmodulin Binding (GO: 0005516). Although Family/control pair shared some significant DMRs overlapping with the DMRs for Cancer/control pair, no pathways and only one *molecular function* survived the FDR on gene ontology tests. This may explain why Cancer group developed CRC while Family group did not.

For obese/control pair, from the 968 DMRs (adjusted *q* < 0.05), we identified 617 known genes. GO analysis resulted only one significant *biological process*, one significant *cellular component* and one significant *molecular function*, named Homophilic Cell Adhesion via Plasma Membrane Adhesion Molecules (GO: 0007156), Plasma Membrane (GO: 0005886) and Calcium Ion Binding (GO: 0005509) respectively. All three categories showed up on Cancer/control pair comparative analysis, indicating that there might be some associations in the development of diseases for CRC and adiposity.

## DISCUSSION

In this study, we determined the methylation status of approximately 25% of CpGs for the entire human genome, using RRBS of blood samples, for the CRC patients and their family members, with the obese and non-obese healthy controls from GEO database. These CpGs were highly enriched in CGI regions. Over 23,000 CGIs were examined in each sample from all four groups. To our knowledge, this is the first sequencing-based methylation study of the blood samples for CRC and their blood-related family members. We also included data from obese and non-obese healthy control participants, accessed from GEO public data to study the common and different epigenetic alterations in CRC and family compared to these controls. After scanning the genome using 200 bp tiling windows, when 10% of differential methylation level was used as criteria, 7327 DMRs were identified between CRC and control pair, 6072 between Family and control pair, and 2203 between obese and non-obese control pair. The decreased numbers of DMRs down the pairs of CRC, Family, obese groups paired with controls, depict the differences of association from CRC, to family members, and obese groups compared to the healthy non-obese group. The findings also suggested that adiposity may share similar disease development in *biological process*, *cellular component,* and *molecular function*, with CRC.

We identified 1778 known genes that were hyper- or hypo-methylated in CRC group when compared to the control. Among the top 10 hyper- or hypo-methylated genes from CRC/control pair, most of DMRs have been reported as being differently expressed in or associated with CRC tumor tissue. *PCNXL3* was reported to be one of the top 20 genes with the highest correlations with colon adenocarcinoma [42], *MIR4285* and *MIR3648* were shown to be differentially expressed in colon adenomas [43, 44], and *MiR1247* as a potential tumor suppressive gene [45]. Bhatlekar et al showed that overexpression of *HOXA4* and *HOXA9* contributes to self-renewal and overpopulation of stem cells in CRC [46]. *HOXA4* has also been reported to be a potential tumor suppressive gene [47–49]. *CLDN23* expression had been shown to be increased in CRC [50] and patients with down-regulation of *CLDN23* was reported to have shorter overall survival [51]. ***GNAS*** mutations had been identified in several tumors of the endocrine system [52]. ***SLC2A1 (GLUT1)*** and ***SLC2A3 (GLUT3*)** genes were involved in glucose and insulin metabolism which played vital role in development of obesity, diabetes and CRC. *MLH3* is a DNA MMR gene, which plays vital role in cancer development. *FANCG* is a DNA repair gene which is a candidate tumor suppressor gene. *RPSA* (also name as *laminin receptor 1*) transcript was shown to being higher in colon carcinoma tissue [53]. Therefore, those genes were involved in CRC and they may qualify to serve as candidate biomarkers for CRC diagnostic or may provide further understanding on CRC development.

Family members may share the genetic heritage and if lived in same household could share the habitat of similar dietary habits, lifestyles and the same environmental agents that could contribute to the same DNA methylation at some level. Therefore, we performed pair-wise comparisons of Cancer/control, Family/control, and obese/control with the methylation status among the four groups of Cancer, Family, obese, and control Five of six significant genes that played significant roles in cancer development or tumorigenesis, were shared by Cancer/control and Family/control pairs as presented in Figures 2 and 3. These genes included ***MSH2, SEPT9, GNAS, SLC2A1/GLUT1*** *and* ***SLC2A3/GLUT*** [20–22], **SLC2A1/*GLUT1*** and ***SLC2A3/ GLUT3***. These genes were associated with poor prognosis for disease-free survival in rectal cancer [24–27], and aggressive CRC through a meta-analysis [28]. ***SEPT9*** gene was reported as being a promising biomarker for early detection and screening of CRC [29, 30], which is also presented in our results using the blood samples. ***CAMK1, GLUT1/SLC2A1*** and ***GLUT3/SLC2A3*** genes were involved in glucose and insulin metabolism that played vital role in development of obesity and diabetes [31].

We further performed GO analysis for both CRC and their family members in comparison to the non-obese healthy controls. However, only one *molecular function* showed significant difference between Family and control pair. Although Family/control pair shared some significant DMRs with Cancer/control pair, no pathways and only one molecular function survived the FDR statistical tests. This may explain the differences on the methylation status associated with cancer development and cancer progression.

For obese and control paired comparison, we presented one significant *biological process*, one significant *cellular component* and one significant *molecular function*, named Homophilic Cell Adhesion via Plasma Membrane Adhesion Molecules (GO: 0007156), Plasma Membrane (GO: 0005886) and Calcium Ion Binding (GO: 0005509) respectively. And these results are repeated components with the Cancer/control paired tests. These shared components demonstrated the potential associations in the development of diseases for CRC and adiposity.

With the conceptualization of family-based study design, in this preliminary report, we have demonstrated the shared genetic and environmental influences, and epigenetics of methylation changes on DMRs and associated genes between CRC and their blood related family members in their genomes. We have also demonstrated the similar methylation changes for the obese subjects in relation to the CRC cases. In addition to many tumor/cancer related genes, we further demonstrated the importance of glucose transporter genes in the methylation pathways for the possible mechanisms of adiposity in promoting cancer progression. Additionally, we demonstrated that the methylation changes can be investigated using the blood samples for the whole human genome as our findings using the blood samples validated findings of tissue-based studies and extended findings from selected genes to the whole genome. Given that this is a first study for CRC cases involving their blood-related family members to examine shared genetic and environmental influences, and epigenetics within the family units, the findings from this demonstration/preliminary project with limited sample size needs to be further validated using larger samples. Our preliminary study provided evidences that these differentially methylated genes in the blood could potentially serve as candidate biomarkers for CRC diagnostic and may provide further understanding on CRC progression. Further studies are warranted to validate these methylation changes for diagnostic and prevention of CRC.

## MATERIALS AND METHODS

### RRBS library preparation

DNA was extracted from the whole blood of human subjects (CRC and their family members) using DNEasy blood and tissue kit (Qiagen, USA), fo1lowing the manufacturer’s protocol. Reduced representation bisulfite sequencing (RRBS) was performed on these DNA samples to identify genomic DNA methylation regions. On the RRBS process: restriction enzyme was used to digest 5 ng genomic DNA in 200 μL reaction buffer at 37°C overnight. The sticky ends produced by digestion were filled with CG nucleotides, and 3’A overhangs were added. Illumina sequencing adapters (Illumina, CA, USA) with 3’T overhangs, instead of standard adaptors contained in Illumina TruSeq library preparation kit, were ligated onto digested DNA following the manufacturer’s protocols (Illumina TruSeq library preparation kit). Size selection was performed manually on a 3% nusieve agarose gel (Alphatech, New Zealand) to capture insert sizes of 150–250 bp based on previous studies [54]. Efficiency of adaptor ligation and size selection was determined by qualitative PCR. Bisulfite conversion of non-methylated cytosines was performed on 20 μL size-selected fragments using an EZ-DNA bisulfite conversion kit (Zymo, CA, USA) following the manufacturer’s instructions, except for a modification to bisulfite conversion conditions as recommended by Smith et al., 2009 [55]: 99°C for 5 minutes, 60°C for 25 minutes, 99°C for 5 minutes, 60°C for 85 minutes, 99°C for 5 minutes, 60°C for 175 minutes, 6 ×(95°C for 5 minutes, 60°C for 90 minutes). All PCR reactions for RRBS were purified using AMPure XP (Beckman Coulter, Brea, USA), analyzed on a bioanalyzer. The libraries were sequenced on two partial flow cells on an Illumina HiSeq 2000 sequencer and 50 bp paired-end reads.

RRBS data for 5 obese human subjects (GEO run number: SRR4048951, SRR4048952, SRR4048954, SRR4048955, and SRR4048957) and 5 non-obese healthy human subjects (GEO run number: SRR4048943, SRR4048945, SRR4048947, SRR4048948, and SRR4048949) were downloaded from GEO database (https://www.ncbi.nlm.nih.gov/geo/query/acc.cgi?acc=GSE85928). Demographic characteristics of gender were matched between groups of obese/non-obese controls, and with the CRC and Family groups to the extent possible; and oldest age from the available controls on the GEO database were chosen, for pair-wise comparison with CRC cases and family members for further DMR analysis.

### Bioinformatics analysis

The raw sequencing reads were cleaned using FASTQC software (Babraham Institute, Babraham, Cambridgeshire, United Kingdom) prior to alignment. Adapter and low-quality reads with a Phred score value of 20 or less were trimmed with Trim galore, a wrapper script using FASTQC and Cutadapt software. The FastQC analysis was then conducted to ensure that quality measures were met in the remaining reads after trimming. The remaining reads were aligned to the hg19 human reference genome by using Bismark with Bowtie 2 software (Babraham Institute, Babraham, Cambridgeshire, United Kingdom), and BAM file was produced. To increase the reliability of the alignments, the average alignment mapping quality (MAPQ) of the BAM file from Bismark was rechecked using SAMtools software [56]. All remaining samples had a MAPQ score greater than 38, indicating the mismatching possibility being small, *p* < 0.00016. Samtools was used to get mpileup, and PERL scripts were used to determine CpG methylation and non-CpG methylation to estimate the bisulfite conversion efficiency. All methylation information was extracted by Bowtie and transferred to a txt file that could be further analyzed and summarized by R program with Methylkit package [57].

### Detection of DMRs

To determine differences in methylation between groups, the aligned extracted data from above were imported into the free open source R package. Percentage of methylation difference per base were calculated using methylKit [57] and DMCs were identified with a >15% methylation difference and an adjusted *p*-value <0.05. eDMR (extended methylKit) were used to identified DMR with at least one DMC, at least three CpGs, a > 10% on mean methylation difference, and an adjusted *p*-value < 0.05. The 200 bp non-overlapping windows was used to identify DMRs. The windows containing fewer than 5 CpGs were filtered out of the further analysis. The DMRs were annotated using the UCSC RefSeq tracks.

### Gene ontology and pathways analysis

Annotated gene lists were submitted to the David functional annotation database. Significant gene ontology and biological pathways were selected based on Benjamin adjusted *p* (Benjamin *p* <0.05). Student’s *t*-test was used to examine whether methylation levels differed between one of the three groups and the healthy non-obese control group. To correct for multiple testing, the *p* values were checked with FDR and adjusted *p* values.

## SUPPLEMENTARY MATERIALS FIGURES AND TABLES




