# Supplementary material for: Genome wide DNA differential methylation regions in colorectal cancer patients in relation to blood related family members, obese and non-obese controls – a preliminary report
Source: Oncotarget. 2018 May 22;9(39):25557–71. doi: 10.18632/oncotarget.25374 (PMC5986643; doi:10.18632/oncotarget.25374)
Supplement: Supplementary file 1 [file oncotarget-09-25557-s001.pdf]

Genome wide DNA differential methylation regions in colorectal cancer patients in relation to blood related family members, obese and non-obese controls – a preliminary report

SUPPLEMENTARY MATERIALS

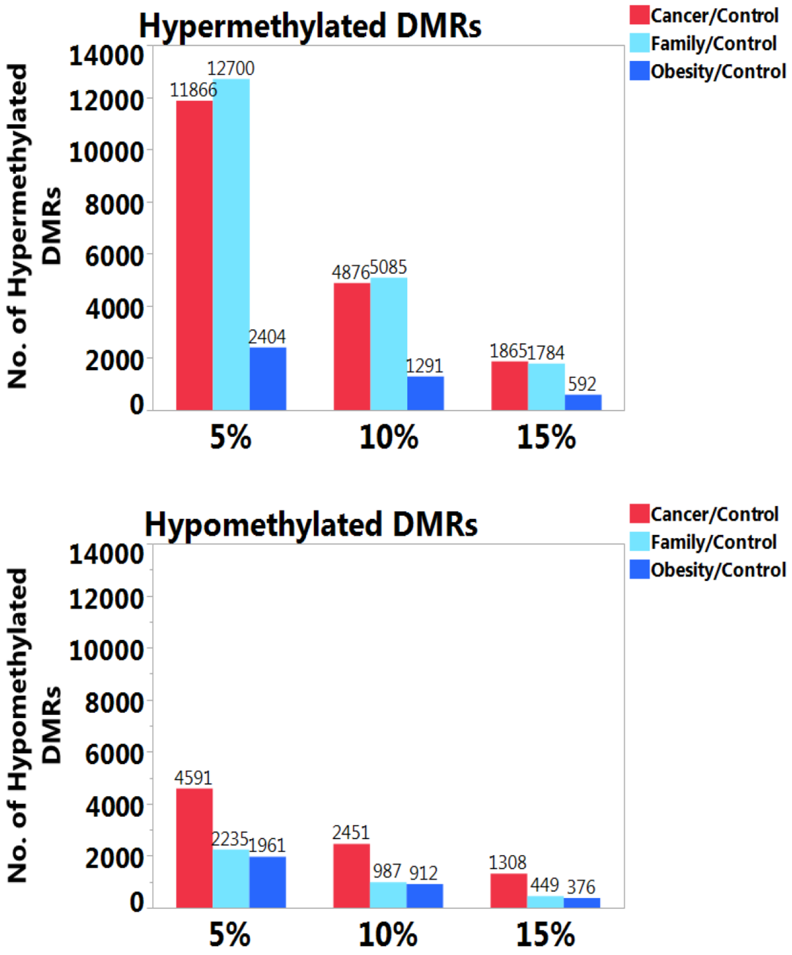

Supplementary Figure 1: Number of hyper-methylation (top) and hypo-methylation (lower) DNA methylation regions (DMRs) identified in samples of Cancer, Family and obese groups when compared with non-obese control samples, with 5%, 10%, and 15% methylation differences between groups.

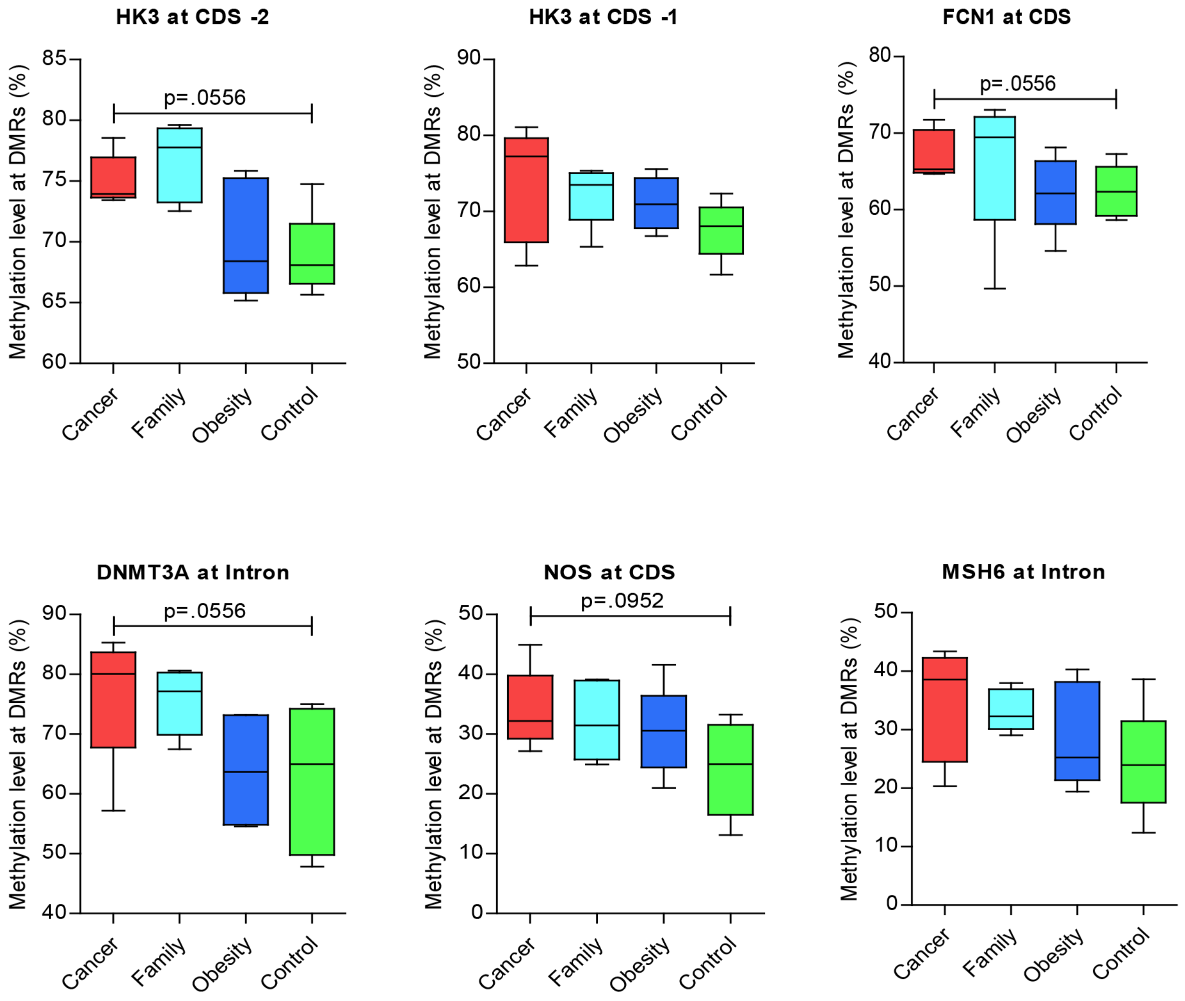

Supplementary Figure 2: Significant genes of hyper-methylation compared between groups of Cancer, Family, and obese with non-obese controls (CDS: coding DNA sequence region).

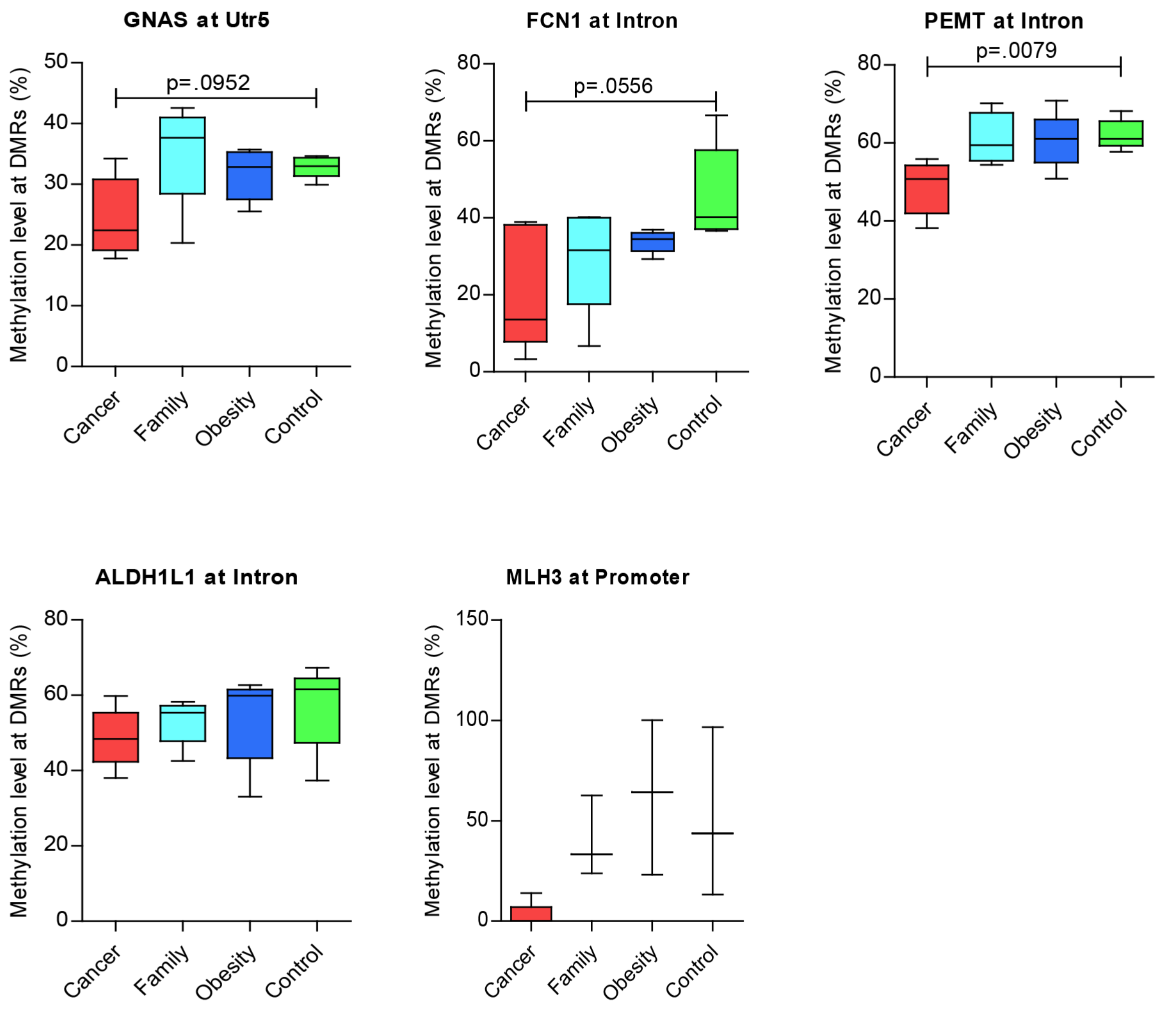

Supplementary Figure 3: Significant genes of hypo-methylation compared between groups of Cancer, Family, and obese with non-obese controls (Utr5: five prime untranslated region).

Supplementary Table 1: Genes listed, full names, and their functions.

See Supplementary File 1

Supplementary Table 2: Distribution of DNA methylation regions based on 10% methylation difference between groups

Groups	Hyper-n DMR (%)	Hypo-n DMR (%)	Totaln DMR %
**Cancer/Control**			
Promoter	272 (5.58)	179 (7.30)	451 (6.16)
Utr5	303 (6.21)	56 (2.28)	359 (4.9)
CDS	1099 (22.54)	203 (8.28)	1302 (17.77)
Intron	1251 (25.66)	871 (35.54)	2122 (28.96)
Utr3	98 (2.01)	39 (1.59)	137 (1.87)
Intergenic	1853 (38.00)	1103 (45.00)	2956 (40.34)
**Family/Control**			
Promoter	211 (4.15)	91 (9.22)	302 (4.97)
Utr5	235 (4.62)	37 (3.75)	272 (4.48)
CDS	1069 (21.02)	81 (8.21)	1150 (18.94)
Intron	1448 (28.48)	317 (32.12)	1765 (29.07)
Utr3	93 (1.83)	24 (2.43)	117 (1.93)
Intergenic	2029 (39.90)	437 (44.28)	2466 (40.61)
**Obese/Control**			
Promoter	96 (7.44)	68 (7.46)	164 (7.44)
Utr5	55 (4.26)	30 (3.29)	85 (3.86)
CDS	198 (15.34)	115 (12.61)	313 (14.21)
Intron	422 (32.69)	266 (29.17)	688 (31.23)
Utr3	33 (2.56)	13 (1.43)	46 (2.09)
Intergenic	487 (37.72)	420 (46.05)	907 (41.17)

Note. Utr5: five prime untranslated; CDS: coding DNA sequence; Utr3: three prime untranslated.

Supplementary Table 3: Top 10 hyper-methylated DNA methylated regions based on 10% methylation difference between Family and Control (-1000 ≤ distance to transcription start site [TSS] ≤ +1000 base-pair DNA)

Gene	Regions	DMR location		Distance To TSS	Methylation Difference			Gene Name or Role
		Start	End		%	*p*	*q*	
PCNXL3	CDS	65,402,926	65,403,007	-774	38.49	7.80E-15	5.85E-13	Homeostasis/ metabolismphenotype
RFPL2	Promoter	32,600,359	32,600,435	284	36.13	3.93E-08	3.85E-07	Metal ion binding
LOC729176	Promoter	147,124,859	147,124,997	0	32.85	3.90E-04	8.75E-04	N/A
TONSL	CDS	145,661,276	145,661,402	-275	31.74	4.04E-09	5.57E-08	Transcription corepressor activity
NLGN2	CDS	7,311,698	7,311,856	198	31.73	1.20E-04	3.32E-04	Mediates cell-cell interactions and modulates insulin secretion
GNAS	Utr5	57,464,110	57,464,426	0	31.72	2.94E-07	2.12E-06	Insulin-like growth factor receptor binding
TPRX1	Intron	48,306,474	48,306,535	327	30.24	8.73E-06	3.73E-05	Sequence-specific DNA binding DNA binding
EGFLAM	Intron	38,446,201	38,446,294	625	30.24	1.28E-06	7.32E-06	Calcium ion binding, glycosaminoglycan binding
PRKAR1B	Intron	601,668	601,797	75	29.77	7.85E-11	1.90E-09	cAMP-dependent protein kinase regulator activity
MIR3648	Utr5	9,825,819	9,826,028	0	29.74	9.88E-21	5.90E-18	microRNA 3648

Note. Utr5: five prime untranslated; CDS: coding DNA sequence.

Supplementary Table 4: Top 10 hypo-methylated DNA methylated regions based on 10% methylation difference between Family and Control (-1000 ≤ distance to transcription start site [TSS] ≤ +1000 base-pair DNA)

Gene	Regions	DMR location		Distance To TSS	Methylation Difference			Gene Name or Role
		Start	End		%	*p*	*q*	
SLC2A3	Intron	8,087,820	8,087,905	988	-55.89	3.22E-10	6.41E-09	Glucose Transmembrane Transporter Activity
LOC338817	Promoter	11,700,194	11,700,609	-355	-46.67	2.61E-19	9.41E-17	N/A
SLC2A1	Promoter	43,425,368	43,425,715	-522	-37.05	3.15E-06	1.57E-05	Glucose TransmembraneTransporter Activity
METTL16	Promoter	2,415,618	2,415,750	-419	-31.59	1.49E-10	3.33E-09	Methyltransferase Activity, RNA Binding
SEPT9	Intron	75,449,408	75,449,544	-531	-31.27	3.90E-08	3.82E-07	Tumor Suppressor Gene
MEG3	Promoter	101,292,199	101,292,350	-95	-30.51	3.13E-15	2.80E-13	LncRNA-mediated Mechanisms of Therapeutic Resistance
HMHA1	Intron	1,077,215	1,077,676	584	-28.99	1.80E-05	6.84E-05	Minor HistocompatibilityProtein HA-1
HOXB6	Intron	46681306	46681384	951	-28.39	8.70E-05	2.53E-04	Transcription Factor activity, Sequence-Specific DNA Binding
CPOX	Promoter	98,313,039	98,313,240	-585	-27.84	1.61E-08	1.82E-07	Porphyrin and Chlorophyll Metabolism
SLC23A1	Utr5	138,718,913	138,719,091	0	-27.55	1.50E-04	3.97E-04	Metabolism of Water-Soluble Vitamins and Cofactors

Note. Utr5: five prime untranslated.

Supplementary Table 5: Top 10 hyper-methylated DNA methylated regions based on 10% methylation difference between Obese and Control (-1,000 ≤ distance to transcription start site [TSS] ≤ +1,000 base-pair DNA)

Gene	Regions	DMR location		Distance To TSS	Methylation Difference			Gene Name or Role
		Start	End		%	*p*	*q*	
RGPD5	Intron	113,190,539	113,190,582	526	62.767	1.4E-08	1.4E-06	RNA Transport
RGPD8								RNA Transport
CTDSPL2	Promoter	44,718,395	44,718,612	-967	33.15	8.31E-10	1.36E-07	Phosphoprotein Phosphatase Activity
GCNT1	Utr5	79,093,253	79,093,300	0	29.59	2.80E-06	8.27E-05	Metabolism of Proteins
LMO2	CDS	33,914,801	33,914,820	-966	29.26	1.48E-08	1.41E-06	Transcriptional Mis-regulation in Cancer
PGPEP1L	CDS	99,548,846	99,549,047	0	27.15	5.99E-06	1.48E-04	Peptidase Activity, Hydrolase Activity
LDHA	Promoter	18,415,098	18,415,164	-772	27.04	2.62E-06	7.89E-05	Glucose metabolism
CYB5R2	Promoter	7,695,499	7,695,680	-26	26.82	7.24E-04	3.84E-03	Amino Sugar and Nucleotide Sugar Metabolism
SPACA1	Utr5	88,757,352	88,757,741	0	26.09	1.30E-02	2.43E-02	Sperm-egg Binding and Fusion
PARVG	Utr5	44,568,741	44,568,932	0	26.02	1.52E-03	6.17E-03	Focal Adhesion
MSH6	Intron	48,011,515	48,011,896	423	25.69	3.43E-02	4.64E-02	DNA Mismatch Repair

Note. Utr5: five prime untranslated; CDS: coding DNA sequence.

Supplementary Table 6: Top 10 hypo-methylated DNA methylated regions based on 10% methylation difference between Obese and Control (-1,000 ≤ distance to transcription start site [TSS] ≤ +1,000 base-pair DNA)

Gene	Regions	DMR location		Distance To TSS	Methylation Difference			Gene Name or Role
		Start	End		%	*p*	*q*	
LHX6	CDS	124,988,308	124,988,968	898	-39.71	5.05E-06	1.28E-04	Transcription Factor Activity, Sequence-Specific DNA Binding
INPP5F	Promoter	121,578,083	121,578,174	-595	-33.92	8.57E-03	1.85E-02	Inositol Phosphate/ PI Metabolism
LHX6	Utr5	124,989,541	124,989,856	10	-33.47	6.97E-10	1.16E-07	Transcription Factor Activity, Sequence-Specific DNA Binding
HIGD1A	Promoter	42,846,923	42,847,117	-897	-30.02	1.68E-05	3.08E-04	CDK-mediated Phosphorylation and Removal of CDC6
SLC2A3	Intron	8,087,820	8,087,905	988	-28.51	1.33E-02	2.47E-02	Glucose Transmembrane Transporter Activity
BLCAP	Intron	36,149,943	36,150,103	337	-26.84	1.94E-02	3.16E-02	Bladder Cancer Associated Protein
NNAT								Regulation of Ion Channels
MATR3	Intron	138,610,352	138,610,539	913	-25.90	3.74E-03	1.10E-02	Nucleic Acid/RNA/ Protein binding
SNHG4								Small Nucleolar RNAHost gene 4
CCDC144B	Utr3	18,528,494	18,528,620	311	-25.28	2.82E-03	9.14E-03	Coiled-Coil Domain Containing 144B (Pseudogene)
DTX1	Promoter	113,495,335	113,495,486	-176	-25.17	1.11E-02	2.19E-02	Notch Signaling Pathway
SSU72	Promoter	1,511,100	1,511,192	-839	-25.17	3.20E-03	9.99E-03	Chromatin Regulation/ Acetylation

Note. Utr5: five prime untranslated; CDS: coding DNA sequence; Utr3: three prime untranslated.
