# Supplementary material for: Genome wide DNA differential methylation regions in colorectal cancer patients in relation to blood related family members, obese and non-obese controls – a preliminary report
Source: Oncotarget. 2018 May 22;9(39):25557–71. doi: 10.18632/oncotarget.25374 (PMC5986643; doi:10.18632/oncotarget.25374)
Supplement: Supplementary file 2 [file oncotarget-09-25557-s002.docx]

Supplementary Table S1. Genes listed, full names, and their functions.

| **Gene** | **Full Name** | **Key Function** |
| --- | --- | --- |
| *APC* | Adenomatous polyposis coli | encodes a tumor suppressor protein, defects cause familial adenomatous polyposis |
| *MGMT* | O-6-methylguanine-DNA methyltransferase | encodes a DNA repair protein involved in cellular defense against mutagenesis and toxicity from alkylating agents |
| *MLH1* | mutL homolog 1 | hereditary nonpolyposis colon cancer (HNPCC). a human homolog of the E. coli |
| *RASSF1A* | Ras association domain family member 1 | encodes a protein similar to the RAS effector proteins. Loss or altered expression with the pathogenesis of cancer |
| *SYNE1* | synaptic nuclear envelope protein 1 | associated with autosomal recessive spinocerebellar ataxia 8, <https://www.ncbi.nlm.nih.gov/gene/23345> |
| *FOXE1* | forkhead box E1 | encoded protein functions as a thyroid transcription factor that plays a role in thyroid morphogenesis. |
|  |  |  |
| *PCNXL3* | pecanex homolog 3 | associated with lipid levels, <https://www.ncbi.nlm.nih.gov/gene/399909> |
| *MIR4285* | microRNA 4285 | short (20-24 nt) non-coding RNAs, <https://www.ncbi.nlm.nih.gov/gene/?term=MIR4285> |
| *NLGN2* | neuroligin 2 | involved in the formation and remodeling of central nervous system synapses, <https://www.ncbi.nlm.nih.gov/gene/57555> |
| *MIR3648* | microRNA 3648 | short (20-24 nt) non-coding RNAs, <https://www.ncbi.nlm.nih.gov/gene/100500862> |
| *HOXA4* | homeobox A4 | encodes a DNA-binding transcription factor which may regulate gene expression, morphogenesis, and differentiation |
| *CLDN23* | claudin 23 | plays a major role in tight junction-specific obliteration of the intercellular space, through calcium-independent cell-adhesion activity |
| *TONSL* | tonsoku like, DNA repair protein | encoded protein is thought to be a negative regulator of NF-kappa-B mediated transcription |
| *GNAS* | guanine nucleotide-binding protein G(s) sub-unit alpha isoforms short | GNAS mutation associated with colorectal tumorigenesis, <https://www.ncbi.nlm.nih.gov/gene/2778> |
| *TUBB8* | tubulin beta 8 class VIII | has a key role in meiotic spindle assembly and oocyte maturation |
| *MIR1247* | microRNA 1247 | short (20-24 nt) non-coding RNAs |
|  |  |  |
| *SLC2A3 (GLUT3)* | solute carrier family 2 member 3 (glucose transporter member 3) | mediates the uptake of glucose, 2-deoxyglucose, galactose, mannose, xylose and fucose, and probably also dehydroascorbate; transport of glucose across the plasma membranes of mammalian cells |
| *LOC338817* | Locus 338817 | with no known function |
| *MLH3* | mutL homolog 3 | DNA mismatch repair (MMR) genes, hereditary nonpolyposis colorectal cancer type 7 (HNPCC7) |
| *LRRC27* | leucine rich repeat containing 27 | associated with lipid levels <https://www.ncbi.nlm.nih.gov/gene/76612> |
| *FANCG* | fanconi anemia complementation group G | encodes a DNA repair protein, candidate tumor suppressor gene |
| *RPSA* | ribosomal protein SA | increased in colon carcinoma tissue and lung cancer cell lines, associated with invasive and metastatic phenotype |
| *SLC2A1*  *(GLUT1)* | solute carrier family 2 member 1 (glucose transporter member 1) | facilitates the transport of glucose across the plasma membranes of mammalian cells |
| *HMHA1* | histocompatibility (minor) HA-1 | <https://www.ncbi.nlm.nih.gov/gene/102950531> |
| *ARID5B* | AT-rich interaction domain 5B | required for adipogenesis, regulates triglyceride metabolism in adipocytes by regulating expression of adipogenic genes |
|  |  |  |
| *RFPL2* | ret finger protein like 2 | <https://www.ncbi.nlm.nih.gov/gene/10739> |
| *LOC729176* | locus 729176 | no known function |
| *TPRX1* | tetrapeptide repeat homeobox 1 | encodes DNA-binding proteins, involved in early embryonic development |
| *EGFLAM* | EGF like, fibronectin type III and laminin G domains | <https://www.ncbi.nlm.nih.gov/gene/133584> |
| *PRKAR1B* | cAMP-dependent protein kinase type I-beta regulatory subunit | cause neurodegenerative disorder |
|  |  |  |
| *METTL16* | methyltransferase like 16 | <https://www.ncbi.nlm.nih.gov/gene/79066> |
| *SEPT9* | septin 9 | candidate for tumor suppressor gene |
| *MEG3* | maternally expressed 3 | is a lncRNA tumor suppressor, <https://www.ncbi.nlm.nih.gov/gene/55384> |
| *HOXB6* | homeobox B6 | associated with acute myeloid leukemia and colorectal cancer, <https://www.ncbi.nlm.nih.gov/gene/3216> |
| *CPOX* | coproporphyrinogen oxidase | encoded enzyme catalyzes the stepwise oxidative decarboxylation of coproporphyrinogen III to protoporphyrinogen IX, a precursor of heme |
| *SLC23A1* | solute carrier family 23 member 1 | encodes sodium-dependent vitamin C transporter |
|  |  |  |
| *RGPD5* | RANBP2-like and GRIP domain protein 5 | arose from a duplication event 3 Mb distal to RANBP2 |
| *RGPD8* | RANBP2-like and GRIP domain protein 8 | <https://www.ncbi.nlm.nih.gov/gene/727851> |
| *CTDSPL2* | carboxy-terminal domain (RNA polymerase II polypeptide A) small phosphatase like 2 | unique chromatin-associated in silenced gene regions and may participate in gene regulation during erythroid differentiation |
| *GCNT1* | glucosaminyl (N-acetyl) transferase 1, core 2 | a member of the beta-1,6-N-acetylglucosaminyltransferase gene family |
| *LMO2* | LIM domain only 2 | has a central and crucial role in hematopoietic development and is highly conserved |
| *PGPEP1L* | pyroglutamyl-peptidase I-like | <https://www.ncbi.nlm.nih.gov/gene/145814> |
| *LDHA* | lactate dehydrogenase A | catalyzes the conversion of L-lactate and NAD to pyruvate and NADH in the final step of anaerobic glycolysis |
| *CYB5R2* | cytochrome b5 reductase 2 | belongs to the flavoprotein pyridine nucleotide cytochrome reductase family of proteins |
| *SPACA1* | sperm acrosome associated 1 | may be involved in sperm-egg fusion, <https://www.ncbi.nlm.nih.gov/gene/81833> |
| *PARVG* | parvin gamma | plays a role in the regulation of cell adhesion and cytoskeleton organization |
| *MSH6* | mutS homolog 6 | DNA mismatch repair gene |
|  |  |  |
| *LHX6* | LIM homeobox 6 | involved in the control of differentiation and development of neural and lymphoid cells, <https://www.ncbi.nlm.nih.gov/gene/26468> |
| *INPP5F* | inositol polyphosphate-5-phosphatase F | potential tumor suppressor in gliomas via inhibition of STAT3 pathway, <https://www.ncbi.nlm.nih.gov/gene/22876> |
| *HIGD1A* | HIG1 hypoxia inducible domain family 1A | <https://www.ncbi.nlm.nih.gov/gene/25994> |
| *BLCAP* | bladder cancer associated protein | involved in tumor suppression by decreasing cell growth through initiating apoptosis |
| *NNAT* | neuronatin | plays a direct and indirect role in diabetes, malformation has the potential to cause a variety of cancers |
| *MATR3* | matrin 3 | mutations are associated with familial amyotrophic lateral sclerosis |
| *SNHG4* | small nucleolar RNA host gene 4 | <https://www.ncbi.nlm.nih.gov/gene/724102> |
| *CCDC144B* | coiled-coil domain containing 144B | Pseudogene, <https://www.ncbi.nlm.nih.gov/gene/284047> |
| *DTX1* | deltex E3 ubiquitin ligase 1 | Encodes positive regulator of the notch signaling pathway |
|  |  |  |
| *DNMT3A* | DNA methyltransferase 3 alpha | encodes protein function in de novo methylation. essential for genetic imprinting |
| *HK3* | hexokinase 3 | phosphorylate glucose to produce glucose-6-phosphate, the first step in most glucose metabolism pathways |
| *FCN1* | ficolin 1 | encodes protein predominantly expressed in the peripheral blood leukocytes, and function as a plasma protein with elastin-binding activity |
| *CAMK1* | calcium/calmodulin dependent protein kinase I | a component of a calmodulin-dependent protein kinase cascade |
| *MSH2* | mutS homolog 2 | tumor suppressor gene and caretaker gene that codes for a DNA mismatch repair (MMR) protein, MSH2 |
| *NOS3* | nitric oxide synthase 3 | has a protective function in the cardiovascular system, which is attributed to NO production |
| *PEMT* | phosphatidylethanolamine N-methyltransferase | converts phosphatidylethanolamine to phosphatidylcholine via three sequential methylations by S-adenosyl methionine (SAM) |
| *ALDH1L1* | aldehyde dehydrogenase 1 family member L1 | loss of function is associated with decreased apoptosis, increased cell motility, and cancer progression |
